# Detecting Short-Term Changes in the Activity of Caries Lesions with the Aid of New Technologies

**DOI:** 10.1007/s40496-015-0050-2

**Published:** 2015-04-17

**Authors:** M. H. van der Veen

**Affiliations:** Department of Preventive Dentistry, Academic Centre for Dentistry Amsterdam (ACTA), University of Amsterdam and VU University, Amsterdam, Gustav Mahlerlaan 3004, 1081 LA Amsterdam, The Netherlands

**Keywords:** Caries assessment, New technologies, Caries activity

## Abstract

This paper discusses the use of new technologies for the assessment of caries and more in particular changes in caries activity. Over the past decades, we have seen a shift from restorative treatment caries to a prevention-driven approach. Also there is a need for shorter and less expensive caries clinical trials. These demand earlier detection of lesions and the monitoring of lesion changes longitudinally in time, which has led to the development of new technologies to aid clinical visual examination. Also clinical visual inspection indices have been refined to fit this purpose. There is a constant flow of technologies emerging and disappearing. This review discusses the merits of recent developments regarding their respective uses for research purposes in testing new caries prevention strategies as well as in clinical caries management in dental private practice. Which technique to choose highly depends on the needed resolution of information.

## Introduction

Over the past decades, we have seen a shift in dentistry from invasive treatment towards prevention-driven treatment [1•, 2–5]. This calls for more versatile caries assessment methods that allow longitudinal follow-up of caries lesions in time [6, 7]. Such methods should be able to detect the onset of caries, or white-spot lesions, when remineralization is still an option and restorative care is not yet needed. Furthermore such methods should be able to determine changes in lesion severity in time. Caries assessment should include the monitoring of changes in the caries activity, where caries activity at subject level is defined as the incidence of new caries lesions and/or the progression of existing caries lesions. At lesion level, active caries lesions have the highest risk of caries lesion transitions among diagnostic categories [8].

Whether for the purpose of caries clinical trials to test the efficacy of new caries preventive agents or for the purpose of caries management in dental private practice, there is a need to assess changes in caries lesion activity due to de- or remineralization as alternative outcome, instead of the cavitation end-point of failed caries prevention [3–10, 11•]. As outcome of the International Consensus Workshop on Caries Clinical Trials (ICW-CCT), held in Loch Lomond in 2002 [6, 7], caries assessment methods need to cover the whole range of caries, from the early white-spot lesion to frank cavitation and be able to detect both caries progression and regression. Simple counting of the number of decayed, missing, or filled teeth (DMFT) or surfaces (DMFS) is too coarse. Changes in dietary pattern or oral hygiene should be reflected in a change in caries activity status. This calls for either monitoring in time, allowing the assessment of changes in lesion severity or assessment of lesion activity at a single point in time.

Current clinical standard of caries assessment is visual or visual-tactile (ball-ended probe) assessment of caries complemented with the use of x-ray photographs. In general only caries incidence and caries progression were scored. In 2005 the International Caries Detection and Assessment System (ICDAS) a 7-point ordinal scale was introduced for the monitoring of caries [12••]. Since then ICDAS has been validated in caries clinical trials [13–17] and aids for the assessment of caries lesion activity have been introduced [18•, 19–23]. Attempts to assess lesion activity have been based on: presence of plaque, location of the lesion at a plaque stagnation site, surface roughness and dehydration pattern of lesions [22–25], or a subjects capability to remineralize an etched tooth area within 24 h [26, 27]. The validity of methods predicting caries progression over time based on a one-time assessment of lesion activity is low for smooth surfaces to moderate for occlusal surfaces [8, 20]. Caries experience may be equally valid as predictor of caries risk [20]. When data from a previous caries examination is available, it is preferred to predict caries activity from changes in time.

## Clinical Visual Caries Assessment

In caries clinical trials, the DMFS or DMFT score is the accepted parameter of disease [28]. Since the introduction of fluoride, the caries levels have declined [29, 30]. The use of fluoride dentifrice has become standard care, and new preventive agents should be tested and proven efficacious against a positive control, e.g., a 1450-ppm sodium fluoride dentifrice [31•]. This calls for a larger number of subjects and/ or longer studies to assess such new caries preventive agents. This implies that costs for running a caries clinical trial have gone up. Therefore caries clinical trials are often run using earlier signs of caries as outcome parameter [31•]. Furthermore one needs to now that new preventive agents not only prevent caries lesion incidence but also promote the remineralization of existing caries lesions. Hence one needs to monitor both progression and regression of caries lesions [6]. The use of clinical scores of caries lesion severity, such as ICDAS, may already provide such a monitoring system [6–7, 12••]. While an ICDAS score is recorded for each surface assessed, a direct comparison of lesions in time is hampered somewhat due to the fact that the lesion is reduced to one categorical number. Any change in lesion extent within the same ICDAS score cannot be detected [32]. In cases where such changes do matter, ICDAS may be complemented by standardized oral photographs, allowing a side-by-side comparison of before and after photographs [32]. Another option is to not only monitor the ICDAS score but also record indicators of caries activity [20].

## Caries Assessment Technologies Based on Optical Properties of Dental Hard Tissues

The majority of caries assessment techniques are based on changes in the optical properties of teeth. These may be based on, e.g., changes in the scattering properties of enamel, translucence, reflectance, and fluorescence using broad-spectrum white light or narrow band short or long wavelength light (violet-blue, red, or (near) infrared). Given the desire to assess short-term changes in caries activity, the use of irradiation techniques will not be discussed. An overview of techniques and their working principles are given in Table 1.

**Table 1:** Overview of caries assessment techniques, aids to visual inspection

Technique	Physical property	Type of result	Measurement modality	Type of surfaces and lesions
Electrical caries monitor	Conductance (1/resistance)	Number [0–99]	Pin-point	Occlusal fissures. Suitable for enamel and dentinal lesions
AC impedance spectroscopy, CarieScan	Impedance	Number [0–99], color signal	Pin-point	Occlusal fissures. Suitable for enamel and dentinal lesions
Scattering monitor, MIDWEST Caries-ID	Backscattering and reflectance	Number (scattering monitor), color and audio signal (Caries-ID)	Pin-point	All accessible surfaces. Only early non-stained enamel caries lesions
FOTI/ DI-FOTI/ DIAGNOCAM	Transillumination	Visual view or image	Visual	FOTI: approximal surfaces only. More than half-way into enamel. DIFOTI: approximal and free smooth surfaces early and advanced lesions
DIAGNOdent (-pen)	Fluorescence	Number [0–99]	Pin-point	All accessible surfaces. Most suitable for advanced lesions
QLF-D, QLF, VistaProof, Spectra	Fluorescence	Image, fluorescence loss [%] and lesion area [pixels] (QLF-D, QLF) or number [1–5] (VistaProof, Spectra). Lesions appear as gray or dark spots, healthy tissue appears white (QLF-D) or green (QLF, VistaProof, Spectra). Lesions may also display red fluorescence	Photographs	All accessible surfaces. Suitable for early caries assessment and advanced lesions. Matured plaque is visible as red fluorescence
Sopro-Life	Fluorescence	Image of fluorescence pattern superimposed on the reflectance image, caries appears as dark shadows and/or red fluorescing areas	Photographs	All accessible surfaces. Suitable for early caries assessment and advanced lesions
OCT	Time of flight backscattering	2D or 3D high-resolution images of cross-section of the tooth structures scanned	Scanning device	All surfaces and lesions. Suitable to detect early demineralization and remineralization, but also the thickness of the remaining layer of dentine in caries excavation
PTR-LUM	Thermal pattern resulting from luminescence	Number [0–99]	Pin-point	All surfaces and lesions

### Scattering

When enamel is demineralized at the earliest stage of caries, this results in a higher scattering coefficient [33, 34]. Hence the earliest signs of caries appear whiter than the surrounding sound tissue. The scattering monitor is a technique based on this principle [35]. Using a fiber-optic probe, light is directed towards the tooth. The back scattered light is retrieved by fibers surrounding the illumination fiber. The intensity of light retrieved in the detection fibers is converted into a number. By scanning over a tooth surface, one may detect white-spot lesions and record the level of decalcification. The need to scan surfaces makes the technique laborious.

The technology has been used in the MIDWEST caries-ID (Dentsply) [36], which uses red and near-infrared LEDs as illumination source. The back scattering ratio of both illumination wavelengths is compared to that of a calibration ceramic to determine health or disease [37]. When demineralization is detected, this is made visible by a red signal, accompanied by beeps at frequencies indicating the demineralization extent, but without means to record lesion severity. The technique is marketed as a detection device and, due to lack of quantification means, is not a monitoring device, making it less useful in determining the success of preventive treatment strategies.

### Fluorescence

Dental hard tissues fluoresce green (510–535 nm) when illuminated with (violet-)blue light in the range of 370–500 nm [38]. Demineralization results in a loss of observed green fluorescence [39]. Red fluorescence is observed in the oral cavity from porphyrin-like metabolic products from bacteria in the dental plaque [40–43]. This red fluorescence can be observed in matured dental plaque, calculus, and caries lesions, from white-spot stage to frank cavities [40, 41, 44, 45]. The red fluorescence is induced by illumination in the blue (∼405) as well as the red part of the spectrum (∼655 nm), with peaks around 630–720 and 700–750 nm, respectively.

Quantitative light induced fluorescence (QLF) is a method initially developed for the detection of early caries lesions by quantification of green fluorescence loss as indirect measure of demineralization [46]. The conventional QLF system (Inspektor Research Systems BV, Amsterdam, The Netherlands) as well as derived systems VistaProof (Dürr dental AG, Germany) and SPECTRA (Air Techniques, Inc. Melville, New York, USA) comprise an intra-oral camera with illumination means in the violet-blue part of the spectrum (∼405 nm) and a high-pass filter in front of the camera sensor blocking the excitation wavelengths and transmitting both green and red fluorescence from the dental tissues. Caries lesions appear as dark areas surrounded by bright green fluorescing healthy tissue, where advanced lesions may also display some red fluorescence. Fluorescence images are assessed for areas of green fluorescence loss and presence and intensity of red fluorescence. While the QLF system provides a continuous scale of green fluorescence loss and lesion area as well as red fluorescence levels, the VistaProof and SPECTRA systems transform green and red fluorescence ratios to an ordinal scale from 0 to 5 reflecting health and severe caries [47, 48]. For adequate monitoring of caries, preferably images should be captured of each tooth surface which can be linked to a patient data management system. While suitable for caries trials, in general practice, one usually only captures images of detected lesions, which can then be followed in time.

To allow imaging of the whole mouth at a glance, the visualization of white-light photos and fluorescence photos side-by-side, and the detection of red fluorescence even in early white-spot lesions, a new QLF camera system was developed (QLF-D, Inspektor Research Systems BV).

This new system uses a single lens reflex camera body (Canon model 450-D or up fitted with a 60-mm Macro lens, Canon Inc., Tokyo) equipped with an illumination and filtering tube (Biluminator Inspektor Research Systems BV, Amsterdam). The Biluminator tube consists of a ring mounted violet-blue LEDs (405 ± 20 nm) and white LEDs (broad spectrum, 6500K) for illumination of the oral cavity, with filtering optics in the optical detection path of the camera, that allows the capturing of both white-light and fluorescence images. The use of differential filtering techniques has resulted in a bright white luminescence of the healthy dentition, while caries lesions appear dark. The red fluorescence signal from matured plaque, calculus, and caries lesions is enhanced in comparison to the conventional green fluorescing QLF images. Quantification of caries lesions results in assessment of fluorescence loss, lesion area, and level of red fluorescence in the lesion. The technique is expected to have similar sensitivity and specificity as its predecessor, but validation of the technique in caries clinical trials needs to prove this.

In 2010, the Sopro-Life (Sopro-Acteon, La Ciotat, France) was introduced which is a fluorescence camera system using both white-light imaging and fluorescence imaging at 450-nm excitation [49, 50]. The fluorescence image is superimposed on the anatomical image of the tooth providing caries details. The system does not provide quantification of caries lesions but relies on visual assessment similar to, e.g., ICDAS. Apart from a daylight mode, also diagnostic and treatment modes are provided. Due to its many options, like magnification and different view modalities, the system seems less suitable for standardized photography of teeth, rendering it difficult to monitor caries over time.

Last but not the least, DIAGNOdent and DIAGNOdent pen (KAVO, Biberach, Germany) are devices using the red fluorescence in caries lesions to assess the level of disease. The red fluorescence is excited by a 655-nm laser. The intensity of red fluorescence around 700 nm is detected and converted into a value between 0 and 99 [51]. The devices are mainly used to scan across the occlusal and/or approximal surfaces but may equally well be used to assess caries on the smooth surfaces. Given the time needed to scan a full mouth, the technique is highly suitable as adjunct to visual inspection. Lesions, once detected, can be monitored in time to judge progression and regression. In comparison to the camera devices, the DIAGNOdent is a less expensive device.

### Transillumination

The translucence of enamel and dentine has been used since the 1980s for the detection of proximal caries. The fiber-optic transillumination (FOTI) technique in its simplest form consists merely of a fiber-optic illuminator using broad spectrum white light, which is held just below the contact-point in the interproximal area [7, 52]. In healthy teeth, the enamel will be uniformly illuminated. When a caries lesion is present in the proximal surface, light will be blocked and a dark area is visible from the occlusal view. Also occlusal caries can be viewed. FOTI is a qualitative technique and as such not suitable to monitor caries in time. The technique has been developed further into a digital imaging system (DI-FOTI) that allowed the observation of not only proximal and occlusal caries but also smooth surface caries, covering the whole range from incipient caries to frank cavitation in all surfaces [53, 54]. Preliminary results on quantification of caries lesions were promising, but the system is no longer on the market. Recently a near-infrared transillumination camera system has been introduced to the market (DIAGNOcam, Kavo) [55]. In this new device, the broad spectrum illumination source has been replaced by a narrow band light source in the near-infrared, which should in theory allow the light to penetrate deeper into the tissue. Only one clinical evaluation study has been published to date. Focus of that study was on detection of proximal and occlusal caries in need of restorative care. They conclude that NIR transillumination may help to avoid bitewing radiographs for diagnosis of caries in everyday clinical practice. Also the manufacturers’ focus seems to be on detection of caries and assessment of restorations for presence of secondary caries.

### Optical Coherence Tomography

The use of optical coherence tomography (OCT) has thus far been limited to research. In vitro research has focussed on the understanding of the caries process and detection of the different stadia [56–58]. Clinically OCT devices have been used to assess sealant efficacy [59, 60], caries on smooth, occlusal, and approximal surfaces [57, 61–64], erosion [65], and cracks [66, 67]. Attempts to introduce OCT systems into the dental clinic have unfortunately been unsuccessful. New developments to improve scanning [68] may pave the way into the dental clinic. Unlike the other optical techniques, OCT produces images of the microstructure of tooth tissues assessed from the outer surface towards the pulp [69•]. OCT thus allows both qualitative and quantitative assessment of caries tooth structure in depth. From the 2D depth scans, a 3D representation can be computed. Unlike the aforementioned techniques, OCT is not affected by plaque or calculus. Given the time required for scanning all surfaces in the oral cavity, OCT is rather used to inspect, e.g., the occlusal surfaces and lesions development under sealants or adjacent to restorations [59, 70, 71]. Lesions detected can be monitored over time by simply comparing images or derived lesion depths.

### Photothermal Radioluminescence

A combination of luminescence and thermal pattern resulting from irradiation with laser light (660 nm) at varying pulse durations can be used to determine a depth profile of a tooth, differentiating healthy and decayed tooth structure [72, 73]. Potentially the photothermal radioluminescence (PTR-LUM) signals could be displayed as 3D image showing the location and extent of the decay. The system on the market (Canary, Quantum Dental Technologies Inc., Canada) transforms the PTR-LUM signals into a number, where higher numbers stand for caries decay.

## Caries Assessment Aids Based on Electro-conductance Properties of Dental Hard Tissues

Caries assessment based on changes in the conductance of dentine and enamel has been introduced in the 1990s [74, 75]. These methodologies have proven especially successful for root caries [76] and occlusal caries [77••]. The most recent development of a device developed for the general dental practitioner is the CarieScan based on impedance spectroscopy ([17]). By recognizing the differences between enamel and dentine structures, this device allowed already early stages of occlusal caries to be detected, especially so-called hidden caries. An overview of techniques and their working principles are given in Table 1.

## Discussion

Depending on the site and type of caries being assessed, the choice for a caries assessment aid may differ. When the monitoring of changes in early caries of white-spot lesions is the main objective, methods based directly or indirectly on the scattering properties of enamel may be the most likely choice. Methods that include the recording of photographs have the benefit that a measure of not only lesion depth or mineral loss is provided but also the lesion area, whereas scanning or pin-point methods such as the DIAGNOdent or scattering monitor only provide a single measure for the surface of the most advanced part of the lesion.

Of all methods discussed, the red fluorescence assessed with the DIAGNOdent, QLF, VistaProof, or QLF-D is considered to be related to metabolic products in plaque and hence in theory an indirect measure of caries activity. The observation that red fluorescence is lost in time may be indicatory of lesion improvement even if the fluorescence loss of the lesion is not changed. In an arrested lesion, the red fluorescence is expected to diminish, but so far, data is merely anecdotal. Whether this hypothesis is true or false needs to be evaluated in a caries clinical trial in subjects with existing active caries using a proven caries prevention method versus a control.

While all caries assessment methods discussed are merely aids to visual assessment, it is a pity that evaluation studies all judge them as a stand-alone technique. Current focus in research has been on detection of caries and determining lesion severity with respect to non-operative or operative treatment needs. This however is and should remain the discretion of the dental professional. The technologies should aid the monitoring of caries lesions, by providing a measure of lesion severity.

Proper assessment of the diagnostic value of techniques is difficult clinically, due to a lack of an adequate reference test, given that most technologies aim at detecting caries lesions earlier than by visual detection. Most diagnostic studies are therefore performed in vitro using histology, microradiography, or similar as a reference test. Perhaps the best validation of a caries assessment technique would be the evaluation of an anti-caries agent with known efficacy in comparison to a control in a caries clinical trial setting [31•]. Few such studies exist. The device most widely used in caries clinical trials is the conventional QLF camera. The system has proven to discriminate between fluoride regimens aimed at remineralization of caries [31•].

It highly depends on research or clinical needs, which technology is best suited. In a study where QLF was used to assess remineralization of white-spot lesions after orthodontic treatment, it was shown that standardized oral photographs were equally useful to detect changes in caries severity and outperformed ICDAS [32]. The expensive and more sophisticated option may not necessarily be the best option. In a high caries risk population, the use of DMFS scores or ICDAS alone may already provide sufficient detail. When caries adjacent to restorative materials is assessed, fluorescence imaging techniques [78, 79] or optical coherence tomography seem the best choice. When high-resolution details are needed for the sake of treating, e.g., deep dentinal caries or crown preparation [80] or when judging the marginal integrity of a restoration, OCT is the technique of choice [69•, 81, 82•].

Perhaps the best part of new technologies is that in clinical practice, they involve the patient in caries assessment and treatment planning. By providing information about oral health status including lesion severity, especially when visualized in photographs, the patients can understand their needs. As such the use of these technologies may aid the communication with the patient and in doing so aid caries prevention.

## Conclusion

The need for early detection devices for caries lesions has led to the introduction of several optical and electro-conductance-based techniques that may aid visual caries assessment. Due to optical properties of teeth and the imperfect remineralization of enamel, the optical properties of a white-spot lesion in theory cannot return to the healthy situation. In real life, lesions will therefore seldom return from ICDAS 1 or 2 to ICDAS 0 or 1 [32]. Using standardized oral photographs may allow the comparison of lesions before and after the implementation of a caries preventive regimen in a qualitative way.

When more detailed information about, e.g., the extent of caries reversal is desired, one may choose any of the mentioned techniques to quantify the lesion at both times. For an overall impression of lesion severity and a careful indication of activity, the red fluorescence from bacterial metabolic products may be indicative, but this has to be proven in caries clinical trials.

## References

[1.•] Vermaire JH, Poorterman JH, van Herwijnen L, van Loveren C. A three-year randomized controlled trial in 6-year-old children on caries-preventive strategies in a general dental practice in the Netherlands. Caries Res. 2014;48:524–33. **RCT showing that a non-operative care treatment protocol is a (cost) effective method to manage and prevent caries**. 10.1159/000358342 24924292

[2] Soderstrom U, Johansson I, Sunnegardh-Gronberg K (2014). A retrospective analysis of caries treatment and development in relation to assessed caries risk in an adult population in Sweden. Bmc Oral Health.

[3] Kidd E, Fejerskov O. Changing concepts in cariology: forty years on. Dental update 2013;40:277–8, 80–2, 85–6. 10.12968/denu.2013.40.4.277 23829008

[4] Kidd E (2011). The implications of the new paradigm of dental caries. J Dent.

[5] Ekstrand KR, Christiansen ME (2005). Outcomes of a non-operative caries treatment programme for children and adolescents. Caries Res.

[6] Pitts NB, Stamm JW. International Consensus Workshop on Caries Clinical Trials (ICW-CCT)—final consensus statements: agreeing where the evidence leads. J Dent Res 2004;83 Spec No C:C125-8. 10.1177/154405910408301s27 15286139

[7] Pitts NB. Modern concepts of caries measurement. J Dent Res 2004;83 Spec No C:C43-7. 10.1177/154405910408301s09 15286121

[8] Nyvad B, Machiulskiene V, Baelum V (2003). Construct and predictive validity of clinical caries diagnostic criteria assessing lesion activity. J Dent Res.

[9] Zero DT, Fontana M, Martinez-Mier EA, Ferreira-Zandona A, Ando M, Gonzalez-Cabezas C, et al. The biology, prevention, diagnosis and treatment of dental caries: scientific advances in the United States. J Am Dent Assoc 2009;140:25 s-34s. 10.14219/jada.archive.2009.0355 19723928

[10] Zandona AF, Ando M, Gomez GF, Garcia-Corretjer M, Eckert GJ, Santiago E (2013). Longitudinal analyses of early lesions by fluorescence: an observational study. J Dent Res.

[11.•] Pretty IA, Ellwood RP. The caries continuum: opportunities to detect, treat and monitor the re-mineralization of early caries lesions. J Dent. 2013;41:S12–21. **This review discusses the importance and validity of monitoring changes in caries development, for the assessment of oral care products in caries clinical trials**. 10.1016/j.jdent.2010.04.003 23985434

[12.••] Pitts NB, Ekstrand KR, ICDAS Foundation. International Caries Detection and Assessment System (ICDAS) and its International Caries Classification and Management System (ICCMS)—methods for staging of the caries process and enabling dentists to manage caries. Community Dent Oral. 2013;41:e41–52. **This paper discusses the international caries detection and assessment system and its associated International Caries Classification and Management System, i.e. the current standard for visual examination of caries and staging the caries process, which is considered the basis for adequate management of caries**. 10.1111/cdoe.12025 24916677

[13] Sitthisettapong T, Phantumvanit P, Huebner C, DeRouen T (2012). Effect of CPP-ACP paste on dental caries in primary teeth: a randomized trial. J Dent Res.

[14] Singh M, Papas A, Vollmer W, Bader J, Laws R, Maupome G (2013). Predictors of coronal caries progression in adults: results from the prevention of adult caries study. Community Dent Oral.

[15] Zandona AF, Santiago E, Eckert GJ, Katz BP, de Oliveira SP, Capin OR (2012). The natural history of dental caries lesions: a 4-year observational study. J Dent Res.

[16] Zandona AF, Santiago E, Eckert G, Fontana M, Ando M, Zero DT (2010). Use of ICDAS combined with quantitative light-induced fluorescence as a caries detection method. Caries Res.

[17] Teo TKY, Ashley PF, Louca C (2014). An in vivo and in vitro investigation of the use of ICDAS, DIAGNOdent pen and CarieScan PRO for the detection and assessment of occlusal caries in primary molar teeth. Clin Oral Invest.

[18.•] Tikhonova SM, Feine JS, Pustavoitava NN, Allison PJ. Reproducibility and diagnostic outcomes of two visual-tactile criteria used by dentists to assess caries lesion activity: a cross-over study. Caries Res. 2014;48:126–36. **A comparison of the two most widely used methods to assess caries activity of a lesion: Nyvad criteria and ICDAS II ICDAS II criteria with the Lesion Activity Assessment system**. 10.1159/000353094 24335157

[19] Piovesan C, Ardenghi TM, Guedes RS, Ekstrand KR, Braga MM, Mendes FM (2013). Activity assessment has little impact on caries parameters reduction in epidemiological surveys with preschool children. Community Dent Oral.

[20] Guedes RS, Piovesan C, Ardenghi TM, Emmanuelli B, Braga MM, Ekstrand KR (2014). Validation of visual caries activity assessment: a 2-yr cohort study. J Dent Res.

[21] Braga MM, Martignon S, Ekstrand KR, Ricketts DNJ, Imparato JCP, Mendes FM (2010). Parameters associated with active caries lesions assessed by two different visual scoring systems on occlusal surfaces of primary molars—a multilevel approach. Community Dent Oral.

[22] Braga MM, Ekstrand KR, Martignon S, Imparato JCP, Ricketts DNJ, Mendes FM (2010). Clinical performance of two visual scoring systems in detecting and assessing activity status of occlusal caries in primary teeth. Caries Res.

[23] Braga MM, de Benedetto MS, Imparato JCP, Mendes FM. New methodology to assess activity status of occlusal caries in primary teeth using laser fluorescence device. J Biomed Opt. 2010;15. 10.1117/1.3463007 20799836

[24] Al-Khateeb S, Exterkate RA, De Josselin De Jong E, Angmar-Mansson B, ten Cate JM (2002). Light-induced fluorescence studies on dehydration of incipient enamel lesions. Caries Res.

[25] Baelum V, Machiulskiene V, Nyvad B, Richards A, Vaeth M (2003). Application of survival analysis to carious lesion transitions in intervention trials. Community Dent Oral Epidemiol.

[26] Meller C, Santamaria RM, Connert T, Splieth C (2012). Predicting caries by measuring its activity using quantitative light-induced fluorescence in vivo: a 2-year caries increment analysis. Caries Res.

[27] Meller C, Heyduck C, Tranaeus S, Splieth C (2006). A new in vivo method for measuring caries activity using quantitative light-induced fluorescence. Caries Res.

[28] Mendes FM, Braga MM, Oliveira LB, Antunes JLF, Ardenghi TM, Bonecker M (2010). Discriminant validity of the International Caries Detection and Assessment System (ICDAS) and comparability with World Health Organization criteria in a cross-sectional study. Community Dent Oral.

[29] Buzalaf MA, Pessan JP, Honorio HM, ten Cate JM (2011). Mechanisms of action of fluoride for caries control. Monogr Oral Sci.

[30] Lussi A, Hellwig E, Klimek J. Fluorides—mode of action and recommendations for use. Schweizer Monatsschrift fur Zahnmedizin = Revue mensuelle suisse d’odonto-stomatologie = Rivista mensile svizzera di odontologia e stomatologia / SSO 2012;122:1030–42. 23192605

[31.•] Ellwood RP, Gomez J, Pretty IA. Caries clinical trial methods for the assessment of oral care products in the 21st century. Adv Dent Res. 2012;24:32–5. **The use of new technologies and ‘surrogate’ endpoints for the use in caries clinical trials are discussed that support the therapeutic treatment of caries instead of a restorative approach**. 10.1177/0022034512449464 22899676

[32] Beerens MW, Boekitwetan F, van der Veen MH, Ten Cate JM. White spot lesions after orthodontic treatment assessed by clinical photographs and by quantitative light-induced fluorescence imaging; a retrospective study. Acta Odontol Scand. 2014;1–6. 10.3109/00016357.2014.980846 25423022

[33] Darling CL, Huynh GD, Fried D (2006). Light scattering properties of natural and artificially demineralized dental enamel at 1310 nm. J Biomed Opt.

[34] Zijp JR, ten Bosch JJ, Groenhuis RA (1995). HeNe-laser light scattering by human dental enamel. J Dent Res.

[35] Ogaard B, Ten Bosch JJ. Regression of white spot enamel lesions. A new optical method for quantitative longitudinal evaluation in vivo. American journal of orthodontics and dentofacial orthopedics : official publication of the American Association of Orthodontists, its constituent societies, and the American Board of Orthodontics 1994;106:238–42. 10.1016/S0889-5406(94)70042-7 8074087

[36] Patel SA, Shepard WD, Barros JA, Streckfus CF, Quock RL (2014). In vitro evaluation of Midwest caries ID: a novel light-emitting diode for caries detection. Oper Dent.

[37] Karazivan N. Interproximal tooth defects detection. In: office Upat, editor. United States Patent Application US: DENTSPLY CANADA LTD.; 2008.

[38] Kuhnisch J, Heinrich-Weltzien R (2004). Quantitative light-induced fluorescence (QLF)—a literature review. Int J Comput Dent.

[39] Heinrich-Weltzien R, Kuhnisch J, van der Veen M, De Josselin De Jong E, Stosser L (2003). Quantitative light-induced fluorescence (QLF)—a potential method for the dental practitioner. Quintessence Int.

[40] Buchalla W (2005). Comparative fluorescence spectroscopy shows differences in noncavitated enamel lesions. Caries Res.

[41] Buchalla W, Lennon AM, Attin T (2004). Comparative fluorescence spectroscopy of root caries lesions. Eur J Oral Sci.

[42] Konig K, Schneckenburger H, Hibst R (1999). Time-gated in vivo autofluorescence imaging of dental caries. Cell Mol Biol.

[43] Konig K, Flemming G, Hibst R (1998). Laser-induced autofluorescence spectroscopy of dental caries. Cell Mol Biol.

[44] Lennon AM, Buchalla W, Switalski L, Stookey GK (2002). Residual caries detection using visible fluorescence. Caries Res.

[45] Lennon AM, Attin T, Martens S, Buchalla W (2009). Fluorescence-aided caries excavation (FACE), caries detector, and conventional caries excavation in primary teeth. Pediatr Dent.

[46] Stookey GK. Optical methods—quantitative light fluorescence. J Dent Res 2004;83 Spec No C:C84-8. 10.1177/154405910408301s17 15286129

[47] Jablonski-Momeni A, Heinzel-Gutenbrunner M, Klein SMC (2014). In vivo performance of the VistaProof fluorescence-based camera for detection of occlusal lesions. Clin Oral Invest.

[48] Rechmann P, Charland D, Rechmann BMT, Featherstone JDB. Performance of laser fluorescence devices and visual examination for the detection of occlusal caries in permanent molars. J Biomed Opt. 2012;17. 10.1117/1.JBO.17.3.036006 22502564

[49] Panayotov I, Terrer E, Salehi H, Tassery H, Yachouh J, Cuisinier FJG (2013). In vitro investigation of fluorescence of carious dentin observed with a SoprolifeA (R) camera. Clin Oral Invest.

[50] Tassery H, Levallois B, Terrer E, Manton DJ, Otsuki M, Koubi S (2013). Use of new minimum intervention dentistry technologies in caries management. Aust Dent J.

[51] Lussi A, Hibst R, Paulus R. DIAGNOdent: an optical method for caries detection. J Dent Res 2004;83 Spec No C:C80-3. 10.1177/154405910408301s16 15286128

[52] Gomez J, Tellez M, Pretty IA, Ellwood RP, Ismail AI. Non-cavitated carious lesions detection methods: a systematic review. Community Dent Oral 2013;41:55 − +. 10.1111/cdoe.12021 25180412

[53] Astvaldsdottir A, Ahlund K, Holbrook WP, de Verdier B, Tranaeus S (2012). Approximal caries detection by DIFOTI: in vitro comparison of diagnostic accuracy/efficacy with film and digital radiography. Int J Dentis.

[54] Schneiderman A, Elbaum M, Shultz T, Keem S, Greenebaum M, Driller J (1997). Assessment of dental caries with digital imaging fiber-optic TransIllumination (DIFOTI): in vitro study. Caries Res.

[55] Sochtig F, Hickel R, Kuhnisch J (2014). Caries detection and diagnostics with near-infrared light transillumination: clinical experiences. Quintessence Int.

[56] Manesh SK, Darling CL, Fried D (2009). Polarization-sensitive optical coherence tomography for the nondestructive assessment of the remineralization of dentin. J Biomed Opt.

[57] Nakagawa H, Sadr A, Shimada Y, Tagami J, Sumi Y (2013). Validation of swept source optical coherence tomography (SS-OCT) for the diagnosis of smooth surface caries in vitro. J Dent.

[58] Natsume Y, Nakashima S, Sadr A, Shimada Y, Tagami J, Sumi Y. Estimation of lesion progress in artificial root caries by swept source optical coherence tomography in comparison to transverse microradiography. J Biomed Opt. 2011;16. 10.1117/1.3600448 21806254

[59] Holtzman J, Pharar J, Lee K, Ahn YC, Tucker T, Sabet S, et al. Ability of optical coherence tomography to detect caries beneath commonly used dental sealants. Laser Surg Med 2010:49-. 10.1002/lsm.20963 PMC3369270 20848554

[60] Lammeier C, Li YP, Lunos S, Fok A, Rudney J, Jones RS. Influence of dental resin material composition on cross-polarization-optical coherence tomography imaging. J Biomed Opt. 2012;17. 10.1117/1.JBO.17.10.106002 PMC3460808 23224001

[61] Cara ACB, Zezell DM, Ana PA, Maldonado EP, Freitas AZ (2014). Evaluation of two quantitative analysis methods of optical coherence tomography for detection of enamel demineralization and comparison with microhardness. Laser Surg Med.

[62] Na J, Baek JH, Ryu SY, Lee C, Lee BH (2009). Tomographic imaging of incipient dental-caries using optical coherence tomography and comparison with various modalities. Opt Rev.

[63] Shimada Y, Sadr A, Burrow MF, Tagami J, Ozawa N, Sumi Y (2010). Validation of swept-source optical coherence tomography (SS-OCT) for the diagnosis of occlusal caries. J Dent.

[64] Shimada Y, Nakagawa H, Sadr A, Wada I, Nakajima M, Nikaido T (2014). Noninvasive cross-sectional imaging of proximal caries using swept-source optical coherence tomography (SS-OCT) in vivo. J Biophotonics.

[65] Wilder-Smith CH, Wilder-Smith P, Kawakami-Wong H, Voronets J, Osann K, Lussi A (2009). Quantification of dental erosions in patients with GERD using optical coherence tomography before and after double-blind, randomized treatment with esomeprazole or placebo. Am J Gastroenterol.

[66] Imai K, Shimada Y, Sadr A, Sumi Y, Tagami J (2012). Noninvasive cross-sectional visualization of enamel cracks by optical coherence tomography in vitro. J Endod.

[67] Yoshioka T, Sakaue H, Ishimura H, Ebihara A, Suda H, Sumi Y (2013). Detection of root surface fractures with swept-source optical coherence tomography (SS-OCT). Photomed Laser Surg.

[68] Damodaran V, Vasa NJ (2014). Development of an electro-optically tuned optical coherence tomography system for imaging dental lesions. Conf Proc Ann Int Conf IEEE Eng Med Biol Soc IEEE Eng Med Biol Soc Ann Conf.

[69.•] Hsieh YS, Ho YC, Lee SY, Chuang CC, Tsai JC, Lin KF, et al. Dental optical coherence tomography. Sensors-Basel. 2013;13:8928–49. **This review discusses the theoretical background of OCT and it’s applications and explains why the technique is so versatile**. 10.3390/s130708928 PMC3758630 23857261

[70] Fried WA, Fried D, Chan KH, Darling CL (2013). High contrast reflectance imaging of simulated lesions on tooth occlusal surfaces at near-IR wavelengths. Laser Surg Med.

[71] Holtzman JS, Osann K, Pharar J, Lee K, Ahn YC, Tucker T (2010). Ability of optical coherence tomography to detect caries beneath commonly used dental sealants. Laser Surg Med.

[72] Hellen A, Mandelis A, Finer Y, Amaechi BT (2011). Quantitative evaluation of the kinetics of human enamel simulated caries using photothermal radiometry and modulated luminescence. J Biomed Opt.

[73] Hellen A, Matvienko A, Mandelis A, Finer Y, Amaechi BT (2010). Optothermophysical properties of demineralized human dental enamel determined using photothermally generated diffuse photon density and thermal-wave fields. Appl Opt.

[74] Huysmans MC, Verdonschot EH, Rondel P (1995). Electrical conductance and electrode area on sound smooth enamel in extracted teeth. Caries Res.

[75] Ricketts DN, Kidd EA, Wilson RF (1997). Electronic diagnosis of occlusal caries in vitro: adaptation of the technique for epidemiological purposes. Community Dent Oral Epidemiol.

[76] Wicht MJ, Haak R, Stutzer H, Strohe D, Noack MJ (2002). Intra- and interexaminer variability and validity of laser fluorescence and electrical resistance readings on root surface lesions. Caries Res.

[77.••] Gomez J, Tellez M, Pretty IA, Ellwood RP, Ismail AI. Non-cavitated carious lesions detection methods: a systematic review. Community Dent Oral Epidemiol. 2013;41:54–66. **Systematic review of the performance of detection methods for non-cavitated lesions**. 10.1111/cdoe.12021 25180412

[78] Fontana M, Platt JA, Eckert GJ, Gonzalez-Cabezas C, Yoder K, Zero DT (2014). Monitoring of sound and carious surfaces under sealants over 44 months. J Dent Res.

[79] Markowitz K, Rosenfeld D, Peikes D, Guzy G, Rosivack G (2013). Effect of pit and fissure sealants on caries detection by a fluorescent camera system. J Dent.

[80] Fujita R, Komada W, Nozaki K, Miura H (2014). Measurement of the remaining dentin thickness using optical coherence tomography for crown preparation. Dent Mater J.

[81] Oancea R, Bradu A, Sinescu C, Negru RM, Negrutiu ML, Antoniac I (2015). Assessment of the sealant/tooth interface using optical coherence tomography. J Adhes Sci Technol.

[82.•] Tom H, Simon JC, Chan KH, Darling CL, Fried D. Near-infrared imaging of demineralization under sealants. J Biomed Opt. 2014;19. **This paper shows how OCT can be applied to assess caries progression underneath sealants**. 10.1117/1.JBO.19.7.077003 PMC4103580 25036214

